# Diseases of Abdomen

**Published:** 1906-08-18

**Authors:** 


### DISEASES OF ABDOMEN.

**Suppurative Peritonitis.**—The peritoneum has long been recognised as being one of the most long suffering and tolerant of serous membranes. It is very resistant to infection, and has a most effectual and skilful way of dealing with invading bacteria. A new virtue has recently been ascribed to it, namely, a low absorptive power in diseased conditions.[1] Absorption of toxic substances by the peritoneum in cases of suppurative inflammation of that cavity has generally been thought an important factor in the mortality, but the new view holds that the danger in general peritonitis lies rather in the intestinal paralysis it produces and in the extension of the inflammation to subperitoneal tissues. The toxæmic symptoms would thus be caused by absorption from the lumen of the distended and paralysed gut. This view of the pathological process is not without its bearing on treatment. It is generally recognised that the prognosis in general suppurative peritonitis has been enormously improved of late years, partly owing to the earlier recognition of the condition, and partly owing to improved surgical methods. Among the latter the procedure known as the "toilet of the peritoneum" holds with many surgeons an important place. But if absorption is mainly from the lumen of the gut, relief of the intestinal paralysis and simple drainage of the peritoneal cavity should suffice. As examples of the various opinions held by surgeons, may be quoted on the one hand Barnard, who strongly objects to flushing or washing out the peritoneal cavity (though not for the reasons stated above), and on the other hand Lane, who makes a long incision through almost the entire vertical length of the abdomen, flushes out the belly with warm water, wipes all lymph off the coils of intestine, and brings only the edges of skin together. Pads of gauze are left in under the wound to protect the intestines, and a gauze drain is inserted. The abdomen is tightly bandaged and the muscles are sewn together at a later date when the distension has passed off.

*After Treatment.*—The after treatment of operation cases, which is often left in the hands of the general practitioner, is, at any rate, equal in importance to the operative procedure. A convenient summary of the indications has lately been set out by Bryant.[2] The things to be guarded against are: (1) cardiac failure and collapse, (2) loss of fluid from the tissues, (3) toxæmia, (4) paralysis of the gut, (5) pain, (6) oral sepsis. (1) Cardiac failure is to be met by large doses of strychnine hypodermically, to the extent, if necessary, of producing slight twitchings. As much as $\frac{1}{2}$ grain may be given in the first 18 hours. In another case 35 ♏ of liquor strychninæ were given in less than 12 hours without any symptoms of overdosage. (2) The loss of fluid is a serious matter. Large injections of warm saline, either per rectum or subcutaneously, are always necessary, and where rapid absorption seems imperative, the salt solution must be run into the veins. Barnard runs in salt solution (1 drachm to the pint) at 115° F., by means of two tubes and two stout needles, continuously beneath the skin of the thigh. The vessel containing the fluid is placed a foot above the level of the thighs, and about two pints are run in per hour. Infusions may be given to the extent of 15 to 20 pints in the first 24 hours. (3) The toxæmia is combated mainly by the salt infusions which dilute and eliminate the poisons. (4) For gut paralysis or distension 1 grain of calomel may be given every hour, and after a few hours an enema of olive oil, ol. ricini, or glycerine. As much as 72 grains of calomel have been given in this way. mag. sulph., by the mouth or rectum, may also be tried. (5) Pain should not be met by morphia either before or after operation. This is now generally agreed. If necessary, Bryant advises 10—15 minims of nepenthe per rectum. (6) The dry mouth is not only unpleasant for the patient but positively dangerous. As an antiseptic wash, formalin i♏ to ℥j., resorcin gr. x. to ℥j., or glycerin ℥iij. and chinosol (1 per mille) ℥xij. may be employed. The use of large nutrient enemata is also a valuable adjunct to after treatment, especially, of course, in cases of perforated gastric ulcer. The employment of these large enemata was first advocated by Beddard, who directed that the enema should be run in continuously without squirting or air injection, at body temperature, and should not be allowed to trickle down towards the internal sphincter. In this way the white of three eggs, salt gr. xl., peptonised milk ad ℥ix., or arrowroot ℥ij., raw meat juice ad ℥ix. These may be given alternately with ℥x. salt solution every four hours. If badly retained the salt solution may be given subcutaneously, and the enemata only injected every eight hours. In gastric cases enemata should be continued four or five days, in appendicitis, etc., until the vomiting has quite ceased.

[1] Brit. Med. Jour. Epit., Nov. 25, 1905. No. 340. [2] Clin. Jour., Dec. 27, 1905, p. 170.

